# Development of a Concept for Non-monetary Assessment of Urban Ecosystem Services at the Site Level

**DOI:** 10.1007/s13280-014-0502-2

**Published:** 2014-04-17

**Authors:** Daniel Wurster, Martina Artmann

**Affiliations:** Department for Geography and Geology, University of Salzburg, Hellbrunnerstr. 34, 5020 Salzburg, Austria

**Keywords:** Urban ecosystem services, Non-monetary valuation, Multi-scale framework, Service providing and reducing elements

## Abstract

Determining the performance of ecosystem services at the city or regional level cannot accurately take into account the fine differences between green or gray structures. The supply of regulating ecosystem services in, for instance, parks can differ as parks vary in their land cover composition. A comprehensive ecosystem service assessment approach also needs to reflect land use to consider the demands placed on ecosystem services, which are mostly neglected by current research yet important for urban planning. For instance, if a sealed surface is no longer used, it could be unsealed to improve ecosystem service supply. Because of these scientific shortcomings, this article argues for a conceptual framework for the non-monetary assessment of urban ecosystem services at the site scale. This paper introduces a standardized method for selecting representative sites and evaluating their supply of and demand on ecosystem services. The conceptual design is supplemented by examples of Salzburg, Austria.

## Introduction

The non-monetary assessment of ecosystem services has become a popular research field over the last years to demonstrate impacts of land use changes on the potential of ecosystem service provision (Burkhard et al. 2009; Haase 2009; Kroll et al. 2012) or to support decision makers and policies in nature conservation activities to secure and promote ecosystem service supply (Daily et al. 2009; De Groot et al. 2010). Despite a range of case studies assessing ecosystem services in a non-monetary manner, such as regulating (Jansson and Nohrstedt 2001; Haase and Nuissl 2010), provisioning (Fitzhugh and Richter 2004; Hong et al. 2009), or cultural services (Kliskey 2000; Kaźmierczak 2013), research is still confronted with methodological evaluation problems. The scientific discussion about ecosystem service assessment involves the fundamental debate about how ecosystem structures, processes, properties, functions, and benefits for human well-being are connected with each other (De Groot et al. 2002; van Oudenhoven et al. 2012), and how within such a “cascade-model” proposed by Haines-Young and Potschin (2010) ecosystem services can be given meaningful, standardized, and consistent values.

A further challenge for the non-monetary assessment of ecosystem services is the question of scale. It is argued that developing a standardized evaluation method on a landscape scale is challenging as the spatial resolution suffers from inaccuracy when trying to gather comparable data in different case studies (Kroll et al. 2012). Also on a site level, standardized and accurate data mining is still a challenge. Freeman and Buck (2003) demonstrated the importance of detailed mapping of private gardens in cities as they can provide completely different properties depending on their structural composition. To foster the development of a standardized assessment method and to secure spatial accuracy within the evaluation process by accounting natural components that provide services, the Service Providing Unit concept is argued to be useful (Kontogianni et al. 2010). Service Providing Units (SPUs) were defined on basis of species populations by Luck et al. (2003), which contribute to an ecosystem service. According to Kremen (2005) it is crucial to understand connections between ecosystem services and habitat areas as well as the variability in ecosystem services and habitats to support ecological and economically sustainable decision making. Therefore, Kremen (2005) extended the SPU approach by Luck et al. (2003) and integrated Ecosystem Service Providers (ESPs) to assess interactions between individuals and habitats stressing the importance of functional groups. To improve ecological know-how about ecosystem services and their connection to habitat areas, Kremen (2005) suggests the selection of representative study sites of different scales and to assess inherent ecosystem services by standardized methods. Though a range of studies exist assessing ecosystem services on a SPU level, the selection of the investigation units within these studies is based on very specific selection criteria related to the research questions or local circumstances (e.g., Barthel et al. 2010; Borgström et al. 2012) and do not take into consideration a standardized selection method. In the following approach, the SPU concept will be modified using a multi-scale approach for selecting and mapping representative sites to assess the provision of ecosystem services on a site level. Such a standardized method for site selection and ecosystem services assessment in cities is crucial for the following reasons:

- To facilitate the understanding about which urban structures provide and reduce ecosystem services on a site level,
- To develop a framework to evaluate the supply of and demand for ecosystem services within built-up areas and to identify trade-offs and synergies between urban structures and ecosystem services,
- To provide urban planning with an easy applicable method for identifying sites which can be used for densification and which have to be protected against further sealing,
- To interlink ecosystem service provision on a site level to a regional scale.

For characterizing urban ecosystem services (ES) the contrast of provision of functions by natural ecosystems and need by urban residents are of crucial importance (Bolund and Hunhammar 1999). We suggest using structures for selecting, mapping, and assessing ecosystem services, as species and habitat mapping do not sufficiently capture demand and supply, nor providing and reducing elements. Structures can be tagged with reducing or providing properties according to their supply and urban residents’ demand and are at the same time transferable to other cities. Ecosystem services that provide structures mostly belong to green and blue ones (Bolund and Hunhammar 1999), e.g., deciduous trees and conifers (Leuzinger et al. 2010), bushes and meadows (Mathey et al. 2011), or lakes (Peterson et al. 2003). Gray elements, such as houses or sealed surfaces, usually act as reducing elements. Besides mapping the land cover for ecosystem service assessment the integration of land use in addition to land cover is very important as it describes how the land is used and for what (Breuste et al. 2013) and, therefore, describes the demand.

The degree and composition of land cover and land use described by structures are characteristic in urban structural units, which can be defined by their vegetation types, sealing degrees, built-up areas, and building density. Therefore, ecosystem services assessment can be addressed spatially in relation to urban structural units (Breuste 2009). To select representative sites of structural unit types (such as parks, commercial, high and low densely built-up areas), we follow the assumption that a higher degree of green within an urban structural unit potentially leads to a higher amount of structural diversity. In turn a range of different ecosystem services can be provided when a higher degree of structural diversity within one urban structural unit is given (Naeem et al. 1994).

Based on these theoretical groundings the paper conceptualizes a multi-scale approach for selecting, mapping, and assessing ecosystem services on a site scale integrating examples of the case study city Salzburg (Austria).

## Materials and Methods

### Study Area

The city of Salzburg is located in Austria on the northern fringe of the Alps, in the middle of the Salzburger Basin, and at the river Salzach. Salzburg has about 150 000 inhabitants and is well equipped with public urban green areas: parks (historic gardens, landscaped parks, and small city parks), city mountains, cemeteries, verdant banks of the River Salzach, and some smaller canals and lakes. Furthermore, urban agricultural and forest areas exist that are also used for recreational purposes. The highest degree of urban green areas can be found in the south of Salzburg. The north is characterized by a high degree of sealing and high densely built-up areas.

### Conceptualizing a Multi-Scale Framework

**Fig. 1:**
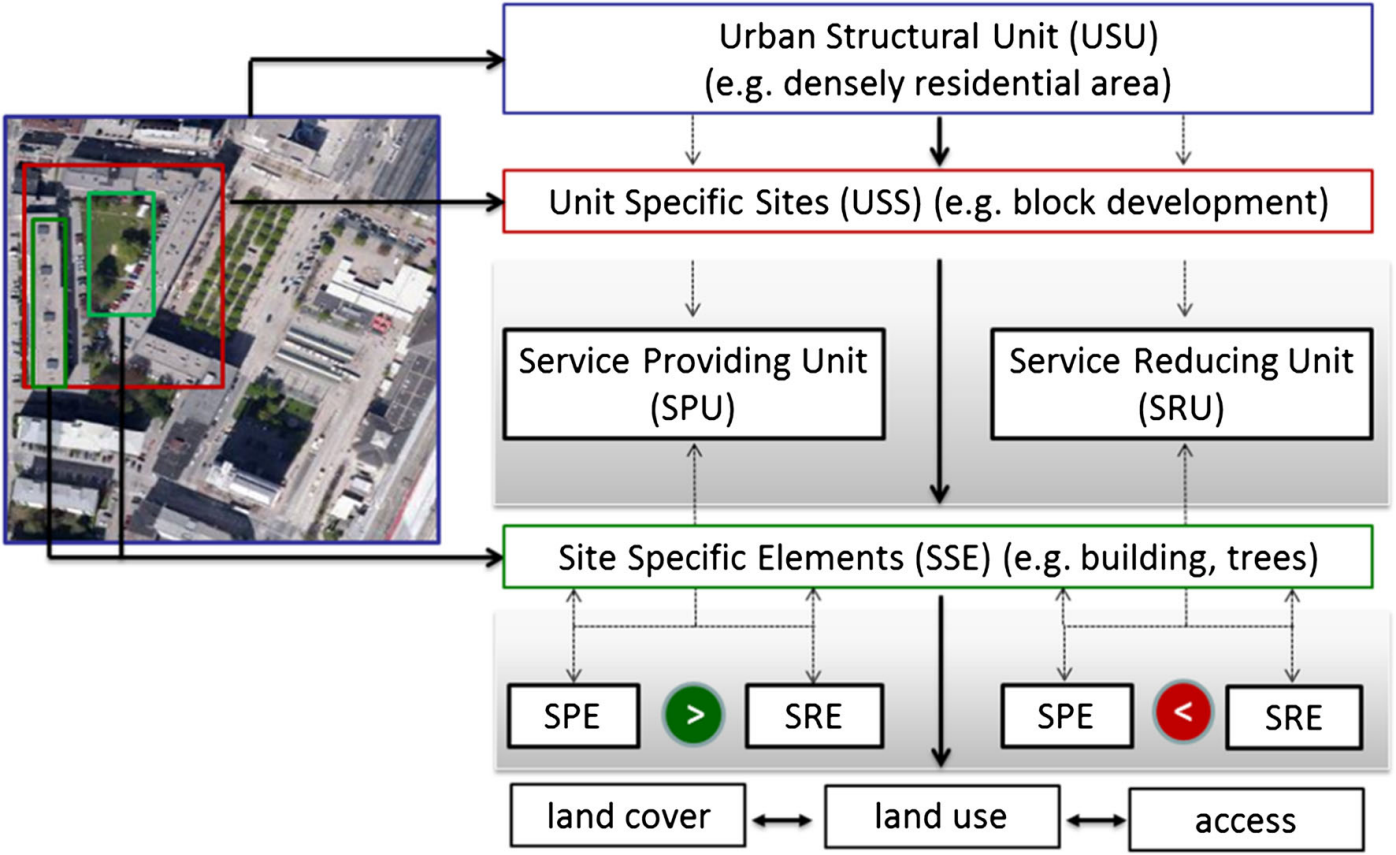
Multi-scale conceptualization for site selection for mapping and assessing ecosystem service provision. *SPE* Service Providing Elements and *SRE* Service Reducing Elements. The *bold arrows* show the vertical and horizontal scales. The *dashed arrows* indicate the compilation of USS by SPEs and SREs which determine whether a USS is a SPU or SRU

The multi-scale approach includes a vertical axis to select representative sites (integrating city to site scale) as well as a horizontal axis (integrating land cover, land use, and access) for selecting, mapping, and non-monetary assessment of multiple ecosystem services. To identify representative structures which are crucial for ecosystem service provision or reduction, three vertical scales are included: urban structural units (USU), unit-specific sites (USS), and site-specific elements (SSEs) (see Fig. 1). A first down-scaling process from a city level integrates urban structural units.

High/low-density built-up residential and commercial sites, agricultural areas, parks, urban forests, and allotments are the focus of this study. These sites were chosen as they represent typical land uses for cities to fulfill the functions of living and working as well as the provision of ecosystem services to various extents. Central to the study are the ecosystem services food production, recreation, and learning about nature as well as microclimate regulation, air purification, and water runoff.

As the units within high- and low-density built-up residential areas and commercial/industrial sites vary in their degree of sealing (Haase and Nuissl 2010) and green volume (Mathey et al. 2011) and USUs within the same types can provide ecosystem services to various extents, these units have been specified in more detail for the unit characteristic land use and land cover types, which will be named as USS, for instance perimeter development or one-family houses.

Looking at the components within the USS the most detailed view analyzes the site characteristic structures, here named as SSEs. These elements can be characterized on the horizontal scale by their type of land cover (asphalt, extensive managed grassland, building with green roof, etc.) as well as by their use (e.g., footpath and green roof with possibility for recreation) and access (e.g., full or conditional access). The type of land cover, use, and access determines whether a specific ecosystem service can be provided (service providing element, SPE) or will be reduced (service reducing element, SRE). At the vertical scale, the composition of the SSEs by SPEs and SREs gives information about whether an urban structural unit is a service providing (SPU) or reducing unit (SRU) (Fig. 1). Therefore, within this framework the SSEs are used as a basis for mapping and assessment.

## Results

### Conceptualization of the Site Selection

To select representative sites which allow a comparative assessment, it is crucial to select sites which differ to a high degree between each other. Since green structures are supposed to be the providers of ecosystem services, the sites have to differ, especially regarding their share of green. Thus, a high and a low share of green point out the sites of interest. Depending on the available data, two different selection methods have been developed.

#### Selection Based on Geo-Information Data

The data used for site selection based geo-information data are from the Space Development Concept Salzburg (REK) and were provided by the City and Federal State of Salzburg. Using already existing data, time and personnel resources can be saved which is crucial, especially for city planning (Larondelle and Haase 2012). Built-up areas (commercial and residential) were grouped into low- and high-density built-up areas as these are seen as being characteristic for urban settlement areas and because of their assumed differences in service provision or reducing potential. Next, representative sites were selected using a raster method and thresholds (Table 1).

**Table 1:** Data and methods for selection urban structural units by geo-informational data

Characteristics of relevance	Data format/source	Use	Method
Cubic index, scales BMZ: till 3.5/3.51–4.1/4.11–6/>6); Green Index GI (degree of green area per grid in %), scales GI: 0–5/>5–25/>25–50/>50–75/>75–95/>95	Shape file cubic index and shape file green index/Regional Development Concept (REK) Salzburg	Define urban structural units of low- and high-density commercial areas and low and high share of green Selection of urban structural units	Putting a grid over map (100 m by 100 m) Intersect cubic index and GI Selection (1) High and low green index in low commercial density (BMZ < 3.5/GI > 75 %; <25 %) (2) Low and high green index in high commercial density (BMZ > 6/GI < 25 %; >75 %)
Floor-space index, SPI (dimensionless), scales: till 0.5/0.51–0.7/0.71–1.1/>1.1); green index (GI) (degree of green area per grid in %), scales: 0–5/>5–25/>25–50/>50–75/>75–95/>	Shape file floor-space index and shape file green index/Regional Development Concept (REK) Salzburg	Define urban structural units of low- and high-density residential areas and low and high share of green Selection of urban structural units	Putting a grid over map (100 m by 100 m) Intersect floor-space and green index Selection (1) High and low green index in low residential density (SPI till 0.5/GI > 75 %; <25 %) (2) Low and high green index in high residential density (SPI > 1/GI < 25 %; >75 %)
Degree of structural diversity in forests (cultivated land use types): beech and mixed woodland, coniferous forests, tree rows and hedges, pedunculate oak and oak-hornbeam forest, pine forests, pioneer and moorland wood, deciduous and commercial forest, commercial wood, and commercial wood addition	Shape file cultivated land use type/Regional Development Concept (REK) Salzburg	Calculation of richness factor for selection of forest plots	Putting a grid over map (400 m by 400 m) Calculating the richness factor per grid via GIS Selection of grids with high and low richness factor
Degree of structural diversity in agricultural areas (cultivated land use types): vegetable fields, horticulture, cereal fields, fodder meadow, root crop, rich pasture, maize fields, bedding meadow, and dry grassland	Shape file cultivated land use type/Regional Development Concept (REK) Salzburg	Calculation of richness factor for selection of agricultural areas	Putting a grid over map (400 m by 400 m) Calculating the richness factor per grid via GIS Selection of grids with high and low richness factor

For the site selection, characteristics of relevance were used which allow evaluation of the potential ecosystem service supply by urban green. The thresholds for grouping low- and high-density built-up areas by the cubic and floor-space index were set to the lowest respective highest value following the assumption that these areas represent low respective high density built-up areas. The threshold for the greening index was set to 25 % respective 75 % due to pragmatic reasons as lower and higher thresholds led to no or fewer sites which, therefore, are not representative. The raster size of 100 by 100 m was used to display a network of sites which can be mapped without requiring too much time and, at the same time, represents the resolution of CORINE land cover data, which allows an up- and down-scaling between the scales. All in all, two-by-two pairs of opposites were selected as representative sites for residential and commercial areas to achieve good and worse examples of green area supply within areas of different built-up densities.

For open spaces the selection was based on a 400 by 400 m raster to include a bigger range of different cultivated land use types. The characteristic of relevance (degree of structural diversity) for the site selection was calculated by a richness factor where sites of the lowest and highest value were selected. All selection steps were carried out using ArcGIS 10 software.

**Fig. 2:**
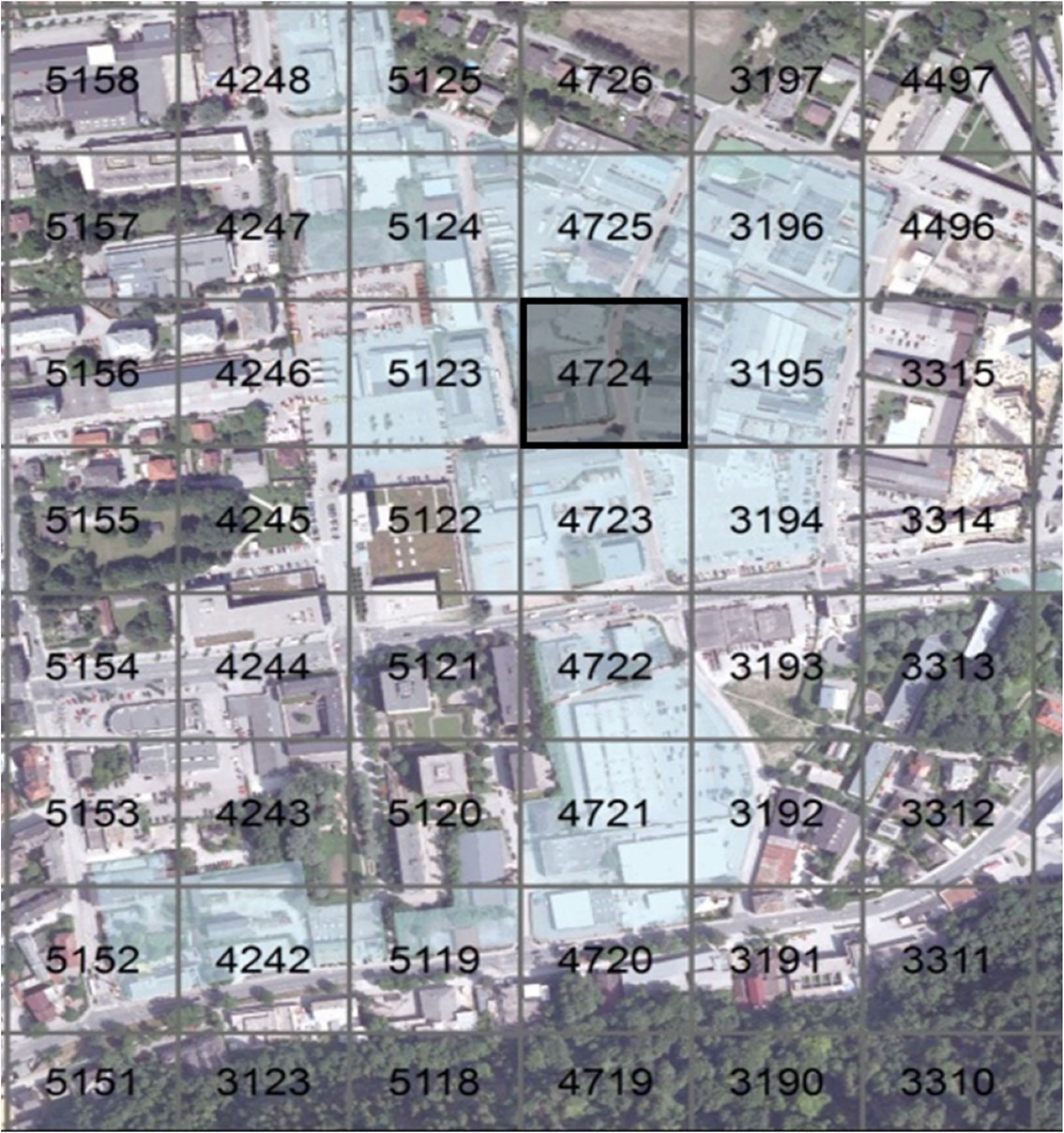
Low dense commercial area with low green index

As an example a commercial site with low density and a low share of green is presented. This commercial site had a high degree of sealing and high space demand and is, therefore, a typical commercial area in Salzburg, and also in other cities. After the intersection of green and cubic index using the thresholds of Table 1, a 100 by 100 m raster was laid on the map. The cell which fulfills the selection criteria best was defined as the core cell. The cells have unique values, which makes it easy to identify exactly one specific cell. Figure 2 shows the selected area that fulfilled the requirements of low density and low green index with the selected core cell 4724. Due to pragmatic restrictions (e.g., cutting a building) the mapping area is defined by clear boundaries (e.g., streets and rivers) surrounding the core cell.

#### Selection Based on Surveys

For parks and allotments no detailed information regarding their structural diversity was available and a site selection by geo-information methods was not possible. To be able to categorize parks and allotments according to their degree of structural diversity a pre-mapping was carried out. Due to their clearly defined shape, parks and allotments need no additional demarcation in the sense of raster cell analysis. To get a first overview of the structural diversity a pre-mapping tool was developed, which identifies a variety of green, blue, and man-made recreational structures which were supposed to provide ecosystem services (Table 2). The pre-mapping was done by proving whether the structure existed or not within the park, where 1 stood for existing and 0 for not existing. All parks bigger than 1 ha and all allotment gardens in Salzburg were pre-mapped. For calculating the structural diversity ratio all green, blue, and recreational elements were summed up and the mean value calculated. From the results the sites with the highest and lowest diversity ratio were chosen for in-depth analysis.

**Table 2:** Range of structures for pre-mapping

Green structures	Blue structures	Recreational structures	
Single trees (>3 species, deciduous/conifers, young/old, with brushwood and tree seedlings)	Lake/fountain (diverse lakeshore yes/no)	Benches	Cafés
Group of trees (>3 species, deciduous/conifers, young/old, with brushwood and tree seedlings)	River/stream/canal (diverse lakeshore yes/no)	Boat rental	Toilets
Forest elements (<3 species/>3 species, coeval, various ages, coniferous, deciduous, and undergrowth)		Playground	bbq-area
Hedges/shrubs (cut/non-cut; <3 species/>3 species)		Dog-place	Open-air cinema
Grassland/lawn (intensive/extensive)		Skate ground	Swimming places
Flowerbed		Bike paths	Others
		Lawn	

### Conceptualization of Mapping Ecosystem Services on a Site Level

#### Requirements for a Standardized Mapping Approach

Urban structural units consist of different SSEs which reflect the properties of a specific area. These elements reduce or provide specific ecosystem services. Due to the development of remote-sensing technologies and geographical information systems (GIS), biotope mapping has improved through quick and accurate mapping methods (Ehlers et al. 2003; Mansuroglu et al. 2006). However, traditional mapping methods incorporate only land cover aspects when mapping and assessing ES using GIS or remote sensing (Troy and Wilson 2006; Burkhard et al. 2009) and disregard use and accessibility aspects of mapped elements. However, as the use of SSEs (e.g., for recreational purposes) by urban dwellers shows up the demand for the element, mapping of use is crucial to evaluate ES rather than just ecosystem functions. The properties and values of SSEs should be easily transferable from literature reviews so no extra measures are necessary. Hence, a mapping key has been developed to identify structures which act either as providing or reducing elements.

#### The Multi-Scale Approach for Mapping Service Providing and Reducing Elements

Based on the requirements for a standardized mapping approach a mapping design has been developed that includes four levels considering the multi-scale conceptualization for site selection (see Fig. 1).

##### Level 1 and 2

The ecosystem service provision within urban structural units and USS (level one) by SSEs is especially determined by land cover (level two) and its degree of sealed surfaces as service reducing elements and amount of urban green and blue areas as the main providers of ecosystem services (Bolund and Hunhammar 1999). The mapping of land cover gives a first assessment basis for the potential ecosystem service provision or reduction.

##### Level 3 and 4

The analysis of land cover lacks a social–ecological connection, which is crucial to understand the relation of multiple ecosystem services (Bennett et al. 2009). Therefore, mapping of SSEs needs to integrate the current land use (third level) and access (fourth level) which are in turn interlinked to the respective land cover of the SSEs identified. The mapping of land use provides information about by whom (e.g., bike and car) and how the SSE is used (e.g., sports, gardening, and no use). The access of blue and green elements is mapped on the basis of Handley et al. (2003) who differentiate five levels of access where 1 stands for full access without any restrictions and 5 for no physical access at all.

##### Example—Mapping Level 2 and 3

**Fig. 3:**
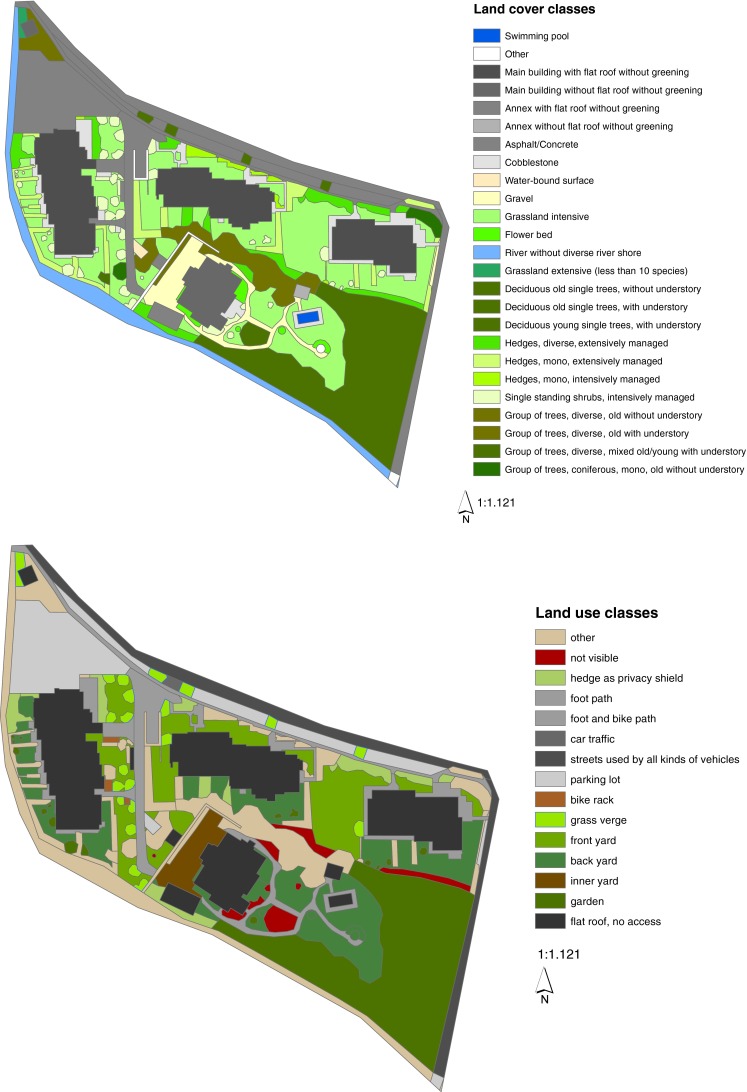
Map of land use and land cover of SSEs of a high density and high green residential area

Figure 3 shows the best practice example in Salzburg of the USU “high density and high green residential area.” The mapping results were digitized in ArcGIS 10 using a high-resolution satellite image. If aspects such as use or accessibility could not clearly be assessed from the satellite image, additional data were gathered during field trips.

### The Concept of Assessing Service Providing and Reducing Elements

**Fig. 4:**
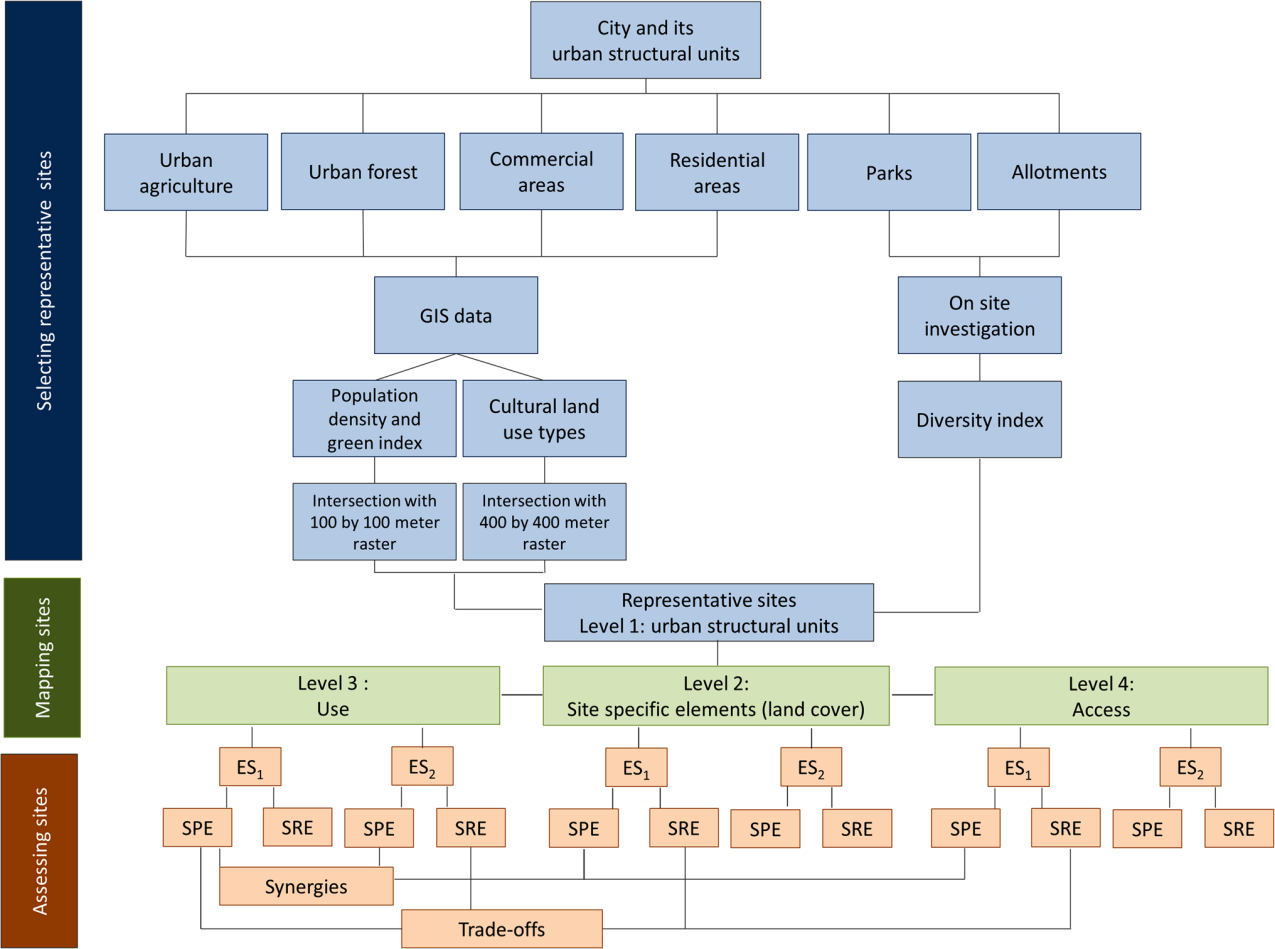
Selection, mapping, and assessment process including possible trade-offs or synergies between different ecosystem services or levels of mapping within an urban structural unit, using the approach of SPEs and service reducing elements (SREs)

Using different scales of mapping, an approach for assessing multiple ecosystem services and their relationships has been developed. These relationships include identifying supply and demand as well as trade-offs and synergies of multiple ecosystem services on a site scale. This seems crucial as the literature lacks standardized approaches for non-monetary assessment of supply and demand, which is particularly difficult on a landscape scale due to lacking spatial accuracy (Kroll et al. 2012) as well as trade-offs and synergies and how to minimize or enhance them, respectively (Bennett et al. 2009). Whether a structural element possesses positive or negative properties regarding the provision of ES strongly depends on the ES in focus. Each element can be, depending on the ES assessed, a reducing or providing element at the same time. Thus, the properties of a SSE might have a positive impact on one ES and a negative on another, which leads to trade-offs. The systematic differentiation between providing and reducing elements allows interpretation of the interrelation between various ES, which in turn offers the opportunity for a strategic optimization of provision dependent on the target (Bennett et al. 2009). These synergies and trade-offs will be addressed within this paper only at a conceptual stage (Fig. 4).

Whether an urban structural unit at the next higher scale becomes a service providing or service reducing unit depends on the normative evaluated composition of its structural elements. Each element is given, (assessing a specific ESS), a value between −1 and 1, where −1 is a maximally reducing property and 1 is a maximally providing property. This value is multiplied by the area the structural element covers. The division of the resulting value through the total area brings up a dimensionless ratio. Depending on the balance between service providing (SPE) and reducing elements (SRE) accounting for a specific ecosystem service (ES), the service provision (SP) by an urban structural unit is determined by whether all urban structural units (USU) have more or less providing elements compared to the amount of reducing elements (Fig. 3). All in all, the SP can be summarized by the equation:

$$ {\text{SP}}({\text{ES}})_{\text{USU}} = \, \sum {\text{SPE}} - \sum {\text{SRE}}. $$

#### Example—Assessing Service Providing and Reducing SSEs

Table 3 shows a sensitive example of how the ecosystem services supply by SSEs within an urban structural unit (USU) can be calculated using the example of microclimate regulation. Due to a high degree of sealed surfaces and lack of green roofs and walls, the unit can be regarded as a service reducing unit for microclimate regulation. In a next step, further indicators showing the demand on/for microclimate regulation can be added and a ratio of supply and demand can be built as in Kroll et al. (2012).

**Table 3:** Example of how to calculate normative values for different SSEs using the example of microclimate regulation in a high density and highly green urban structural unit

SSEs	SSEs cluster	Factor	Area (m^2^)	Value
Swimming pool	Water bodies	1	925.88	925.88
River without diverse river shore				
Main building, flat roof, without greening	Buildings without greening	−1	3097.75	−3097.75
Main building, without flat roof, without greening				
Adjacent building, with flat roof, without greening				
Adjacent building, without flat roof, without greening				
Concrete and asphalt	Sealed surfaces	−1	3275.75	−3275.75
Cobble-stone pavement	Semi-permeable surfaces	−0.5	1067.19	−533.60
Water-bound surface				
Gravel				
Grassland intensive	Grassland intensive	0.2	3443.79	688.76
Grassland extensive, less than 10 species	Grassland extensive	0.3	34.98	10.49
Flowerbed	Flowerbed	0.3	100.31	
Deciduous old single trees, without understory	Deciduous old single trees, without understory	0.7	106.85	74.80
Deciduous old single trees, with understory	Deciduous young single trees, without understory	0.5	53.16	26.58
Deciduous young single trees, with understory	Deciduous young single trees, with understory	0.6	20.85	12.51
Hedges, diverse, extensively managed	Hedges	0.5	1263.07	631.54
Hedges, mono, extensively managed				
Hedges, mono, intensively managed				
Single standing shrubs, intensively managed	Shrubs	0.5	403.38	201.69
Group of trees, diverse, old without understory	Group of trees, diverse, old without understory	0.9	81.95	73.75
Group of trees, diverse, old with understory	Group of trees, diverse, old with understory	1	629.40	629.40
Group of trees, diverse, mixed old/young with understory	Group of trees, diverse, mixed old/young with understory	0.8	2617.40	2093.92
Group of trees, coniferous, mono, old without understory	Group of trees, coniferous, mono, old without understory	0.9	118.33	106.50
Other	Other	0	102.13	0.00
	Sum		17 342.17	−1431.29
	Value USU (ratio value and area)			−0.08

## Discussion

### Use of Different Datasets and Thresholds

Survey areas can be selected by three main approaches: selective, representative, and overall (Schulte et al. 1993; Freeman and Buck 2003). In the methodology presented in this paper, a clear focus on a representative selection has been made as a selective study would not be transferable to other cities and an overall study would be too expensive and time consuming. For the representative study, the decision to choose two disparate examples of a study area, one with a high and the other with a lower structural diversity, was made to assess the potential ecosystem services provision by comparing differences regarding service providing and service reducing properties. The geodata used for the identification of study sites are seen as being appropriate for a standardized methodology. Information on building density, green index, and cultural land types is supposed to be available in most cities. This ensures an applicability of the methodology in planning practice (Larondelle and Haase 2012). In the case of Salzburg, no data were available on the structural diversity of parks and allotments, which made a site selection only possible by a fast mapping of several structural elements within these unit types. The choice of green, blue, and recreational elements for the mapping process has proven to be appropriate as these elements have the potential to provide ecosystem services within these sites. Whether the differentiation between high and less/lower structural diversity leads to a high resp. low ecosystem service supply still has to be proven using the concept of SPR and SRE.

### Use of Different Scales

In the methodology presented in this paper, we integrate vertical and horizontal scales. This leads to a high amount of data and degree of complexity. So far it cannot be clarified if this hinders the applicability in practice and to which degree the complexity might be reduced during different steps of generalization. Nevertheless, we see an importance of integrating land cover, land use, and accessibility to assess ecological and social properties of the sites. In this approach we focus on the potential use of sites and their structural elements, which were derived from satellite image analyses. Such an analysis does not say anything about the current use, which would be more appropriate. Nevertheless, the assessment of the current use by observation or by asking users would be very time intensive and the additional value might be questionable. However, to a certain extent it can be assumed that potential and current uses coincide. Moreover, the potential use obtains an additional value by integrating the accessibility. The integration of a horizontal scale (land cover and use and access of these) allows an interesting possibility to integrate supply and demand of SSEs.

To form a basis for the integration of local and regional scale investigation, the use of representative urban structural units makes it possible to connect assessment and multi-criteria valuation of specific structural elements with their responding urban structural units. Provided that a representative amount of valuations of a specific urban structural unit has been conducted it will be possible, in future studies, to extrapolate local data on ecosystem service assessment to a whole city and region, also using other case study cities.

### Service Providing and Service Reducing Elements

The use of structural elements as indicators has the advantage that, on the one hand, during the mapping process no knowledge of different species is necessary and, on the other hand, these structural elements occur in almost every city. The high level of detail allows assessment of the performances of each SSE and its potential to provide or to reduce ecosystem services thus interlinking measured data with the mapped structures (e.g., infiltration capacity by different coverage types). By normalizing the data of each structure and for each ecosystem service, standardized values for the potential of the structures to provide or reduce services can be developed, which offers the possibility for planners to assess a specific site quickly using a look up table and allows modeling of newly planned areas.

Comparing structural elements and their ability to provide or to reduce ecosystem services allows easily identifiable trade-off or synergies of the respective structure. As the SREs and SPEs are embedded within urban structural units it is possible to assess the ability of the urban structural unit to provide or reduce ecosystem services. Sufficient investigations at this level are required to obtain significant statements and to prove the possible loss of information due to the generalization process.

## Conclusion and Outlook

The conceptual and methodological framework presented in this paper provides a basis for operationalizing the non-monetary assessment of urban ecosystem services on a site level. The framework provides a solution to the problem of comparing data and studies between cities in order to assess ecosystem services. It strikes a balance between detail, accuracy, time, and effort. Moreover, the concept also has the potential to be implemented by the planning practice as data provided by the city were used for selecting the case study sites.

This methodology will next be grounded with data on properties of the SSEs obtained from a literature review regarding the ecosystem services of air pollution reduction, microclimate and water flow regulation, recreation, and learning about nature. Additionally, biodiversity aspects will be integrated, trying to elucidate connections between specific structures and biodiversity. The expected results for the follow-up paper will be a set of standardized indicators for assessing the supply and demand by site-specific elements within the urban structural units presented in this paper. A third paper will address the multi-criteria evaluation to show the current and potential synergies and trade-offs between different services.
